# The unnaturalistic fallacy: COVID-19 vaccine mandates should not discriminate against natural immunity

**DOI:** 10.1136/medethics-2021-107956

**Published:** 2022-03-07

**Authors:** Jonathan Pugh, Julian Savulescu, Rebecca C H Brown, Dominic Wilkinson

**Affiliations:** 1 The Oxford Uehiro Centre for Practical Ethics, University of Oxford, Oxford, UK; 2 Murdoch Childrens Research Institute, Parkville, Victoria, Australia; 3 Newborn Care, Oxford University Hospitals NHS Foundation Trust, Oxford, UK

**Keywords:** COVID-19, public policy, ethics

## Abstract

COVID-19 vaccine requirements have generated significant debate. Here, we argue that, on the evidence available, such policies should have recognised proof of natural immunity as a sufficient basis for exemption to vaccination requirements. We begin by distinguishing our argument from two implausible claims about natural immunity: (1) natural immunity is superior to ‘artificial’ vaccine-induced immunity simply because it is ‘natural’ and (2) it is better to acquire immunity through natural infection than via vaccination. We then briefly survey the evidence base for the comparison between naturally acquired immunity and vaccine-induced immunity. While we clearly cannot settle the scientific debates on this point, we suggest that we lack clear and convincing scientific evidence that vaccine-induced immunity has a significantly higher protective effect than natural immunity. Since vaccine requirements represent a substantial infringement of individual liberty, as well as imposing other significant costs, they can only be justified if they are necessary for achieving a proportionate public health benefit. Without compelling evidence for the superiority of vaccine-induced immunity, it cannot be deemed necessary to require vaccination for those with natural immunity. Subjecting them to vaccine mandates is therefore not justified. We conclude by defending the standard of proof that this argument from necessity invokes, and address other pragmatic and practical considerations that may speak against natural immunity exemptions.

Governments and private enterprises around the world have imposed various vaccine requirements in response to the COVID-19 pandemic. To give a non-exhaustive list of examples, the UK government has proposed making COVID-19 vaccination a condition of employment for care home staff and frontline health and social care workers in England.^1 2^ The US government has imposed a mandate for healthcare workers at government-funded healthcare facilities; however, the Supreme Court blocked a proposed mandate that would have applied to employees at large businesses.^3^ Education workers, construction workers, hospitality workers and athletes in certain jurisdictions have also been subject to vaccine mandates.^4–6^ Citizens in some countries (including France and Israel) must have so-called ‘vaccine passports’ or ‘health passes’ to enter certain public spaces,^7^ while Italy has made its health pass mandatory for all workers.^8^

These measures have been widely debated, with critics raising general concerns about discrimination and human rights^9^; as noted above the US Supreme Court blocked a proposed mandate, and at the time of writing, there is debate about revoking the mandate for healthcare workers in England.^10^ Nonetheless, supporters of mandates have argued that they can be a necessary and proportionate measure for achieving one or both of the following public health benefits: (1) preventing healthcare systems from becoming overwhelmed (by reducing healthcare staff absences and reducing the number of individuals who will experience severe outcomes from COVID-19) and (2) reducing the number of infections in the community by reducing viral transmission.^11 12^ In this paper, we are not concerned with the overall justifiability of these measures; for the sake of argument, we shall assume that they can be justified in at least some form to achieve one or both of these benefits. Given that assumption, we are interested in the issue of the different alternatives and exemptions that these policies admit.

Some vaccine passport schemes (such the Israeli scheme^13^) have also granted passes to unvaccinated individuals who provide either (1) a recent negative test result from stipulated viral detection tests (such as PCR and lateral flow antigen tests) or (2) proof of recent recovery from COVID-19. However, others are stricter; some allow only regular viral detection testing as an alternative to vaccination (such as the US vaccine mandate for healthcare workers), while the strictest mandates (such as the proposed vaccination requirement for healthcare staff in England) do not recognise any testing alternatives to vaccination, and only permit tightly controlled medical (and in some cases religious) exemptions. Policies that do not recognise natural immunity as a sufficient basis for exemption to vaccination requirements (such as the proposed requirement for healthcare staff in England) have attracted particular controversy. Indeed, some organisational policies have already been subject to legal challenges on this point. In recent months, a district court judge upheld the University of California’s decision to not include an exemption for those who could provide proof of natural immunity in its COVID-19 vaccine requirement.^14^ Conversely, according to media reports, George Mason University recently granted a medical exemption to an employee who provided proof of active antibodies following a legal challenge.^15^ Another pending lawsuit involves an academic bioethicist.^16^ Finally, in January 2022, controversy surrounding the tennis player Novak Djokovic’s deportation from Australia focused international attention on the validity of natural immunity exemptions to vaccine requirements for international travellers.^17^

Here, we address the ethical considerations at the heart of these recent controversies, and make the case for recognising proof of natural immunity as an acceptable alternative to proof of vaccination. Our argument shall take a conditional form; *if* vaccine-induced immunity achieves a sufficient public health benefit to justify a vaccine mandate, then such mandates ought to consider evidence of recent infection as a sufficient basis for an exemption. We shall begin by distinguishing this argument from two implausible claims about natural immunity.

## Two false claims about natural immunity and one true claim

The history of the anti-vaccination movement is replete with examples of opposition to vaccination grounded in a concern that vaccines are contrary to nature and compromise purity.^18^ A common trope among the anti-vaccination movement is that natural immunity is therefore superior to ‘artificial’ vaccine-induced immunity. This is a grave mistake and a form of the naturalistic fallacy.^19^ It is ‘natural’ to become immune through contracting infection but it is also natural to die from serious infections.

A second related claim, widely advocated among the anti-vaccination movement, is that it is better to *acquire* immunity through natural infection rather than through vaccination. Rather than suggesting the alleged superiority of natural over vaccine-induced immunity itself (as suggested by the first claim), this second claim relates to the alleged superiority of gaining immunity via a natural rather than artificial process on certain unorthodox understandings of the role of ‘the natural’ in the aetiology of health and disease. Yet, for the vast majority of people, this claim is also patently false, since the risks of serious illness and dying from natural infection are considerably higher than those of vaccination. It would be prudentially irrational to choose to be infected rather than to have the vaccine, for those who are vulnerable to COVID-19. A public health strategy that pursued ‘natural’ herd immunity would lead to vastly higher morbidity and mortality than one that pursued vaccine-induced herd immunity.

However, one need not endorse either of these claims in order to support the claim that we shall defend in the remainder of this paper. This is the idea that, for the purposes of immunity certification, those who have acquired immunity naturally are potentially equivalent to those who have acquired immunity through vaccination. This claim does not depend on either of the problematic and logically flawed views outlined above. Quite the opposite; we suggest that the target of our view—the unquestioning assumption that artificial immunity is superior to naturally acquired immunity for the purposes of certification is itself fallacious—it is a form of ‘unnaturalistic fallacy’.

We shall now briefly survey the evidence base for the claim that natural immunity and vaccine-induced immunity for COVID-19 are broadly comparable.

## Natural immunity and vaccine-induced immunity: an overview of the evidence

The question of the relative effectiveness of vaccine and infection-induced immunity is, of course, an empirical question that is best answered by an appropriate empirical research study, in this case, a systematic review and meta-analysis. Once available, policy decisions should ultimately be guided by this sort of high-quality evidence. However, although one published systematic review of nine clinical studies suggests no statistical advantage to vaccine-induced immunity over natural immunity,^20^ large-scale reviews and analyses (particularly in relation to the most recent Omicron variant) are currently lacking. As such, we shall here provide only an overview of the currently available evidence.

As we detailed in the introduction, there are two benefits of vaccine-induced immunity that are invoked to justify general vaccine mandates: preventing healthcare systems from becoming overwhelmed (in large part by reducing severe outcomes from COVID-19) and the reduction of viral transmission. With respect to the former, large, randomised placebo-controlled studies have clearly demonstrated that widely used vaccines have a high degree of efficacy in preventing serious morbidity and mortality from COVID-19.^21 22^ As time passes, we will continue to learn more about the duration of vaccine-induced immunity, but data suggest that vaccine efficacy declines over time.^23 24^ For this reason, many countries have implemented booster programmes. Notably though, in many countries, individuals are considered to be fully vaccinated for the purposes of vaccine requirements and passport schemes as long as they have received two doses; for instance, a booster does was not necessary for receiving a NHS COVID-19 pass in England while the domestic vaccine passport scheme was in place. However, some countries have imposed expiry dates on the validity of proof of vaccination; for instance, Croatia, Austria and Switzerland consider travellers ‘fully vaccinated’ for only 1 year after their second dose of a COVID-19 vaccine.^25^

The emergence of new virus variants may reduce the efficacy of vaccines. Indeed, following the initial submission of this manuscript, the Omicron variant emerged, and the UK Health Security Agency has closely monitored reports about the impact of Omicron on vaccine effectiveness.^26^ At the time of writing, the latest data suggest that in adults aged 65 or over, there is ‘minimal or no effect against mild disease with the Omicron variant from 20 weeks after the second dose of either a ChAdOx1-S or BNT162b2 primary course’. However, the same report estimated that, following booster doses in this cohort, vaccine effectiveness against hospitalisation is 94% 2–9 weeks after the booster dose and 89% at 10 weeks after the booster dose.^27^

We are also learning more about the effect of the vaccines on viral transmission.^28 29^ Prior to the emergence of the Omicron variant, evidence suggested that the vaccines had some efficacy in preventing infections, as well as symptomatic disease.^30 31^ One study suggested that full immunisation with mRNA vaccines was 90% effective in preventing SARS-CoV-2 infections in real-world settings, regardless of symptom status.^32^ Furthermore, infection survey data from the Office of National Statistics in the UK suggested that full vaccination reduced the risk of testing positive by 79% during the Alpha-dominant period and by 67% during the Delta-dominant period.^33^ These studies are consistent with the claim that the vaccines were somewhat effective in reducing transmission, but there is still a need for clinical trials and observational studies to firmly establish this. Indeed, data are beginning to emerge which suggest that the effect of the vaccines on transmission may diminish within a matter of months, and that they may have less of an effect on reducing transmission.^29 34^ Furthermore, the emergence of Omicron has muddied these waters. At the time of writing, evidence suggests that Omicron is a substantially more transmissible variant^35^; yet, there is also some early preprint evidence to suggest that there may yet be some reduced transmissibility of Omicron in booster-vaccinated individuals compared with individuals vaccinated with two doses (OR=0.72).^36^

Data about the waning of vaccine immunity and Omicron’s potential for vaccine evasion raise a significant challenge for the justification of vaccine mandates (ie, they question whether vaccine mandates are ethical overall). However, these questions need not concern us here. Rather, the key question for our purposes here is whether, *if* a vaccine mandate is being applied, natural immunity would also achieve the public health benefits that are desired. Prior to the emergence of Omicron, a review in the *BMJ* highlighted substantial evidence to suggest that natural immunity confers a comparable degree of protection to vaccine-induced immunity.^37^ Studies found a durable immune response in individuals 8 months after infection,^38^ as well as low infection rates among those who have previously had COVID-19, with data suggesting that prior COVID-19 infection had a protective effect of 81.8% against reinfection (defined as infection ≥90 days after initial testing).^39^

Data also suggested that the antibodies elicited by vaccination have less potency and breadth than those generated by natural infection, although the overall neutralising potency of plasma is greater following vaccination.^40^ Prior to Omicron, this picture was beginning to find further support in population level data. In addition to SIREN study data from January 2021, which showed that a history of SARS-CoV-2 infection was associated with an 83% lower risk of infection,^41^ infection survey data from the Office of National Statistics suggested that prior infection reduced the risk of testing positive by 65% in the Alpha-dominant period and by 71% in the Delta-dominant period (compared with the 79% and 69% risk reduction associated with full vaccination in the respective periods reported above).^33^ Similarly, a preprint study using a large database including the entire adult population of Israel (6.4 million people) found similar protection (in the range of 92.8%–94.8%) against COVID-19 infection and hospitalisation in those receiving COVID-19 immunisation and those who had prior COVID-19 infection.^42^ Population data elsewhere further supported the protective effect of natural immunity.^43–45^ Finally, although data suggest that vaccination is still beneficial in those with natural immunity,^46^ and indeed may achieve the maximal level of protection, data suggest that the absolute reduction in risk that vaccination achieves in those with natural immunity may be small. One large study found that 767 individuals with natural immunity needed to be vaccinated to prevent one reinfection during follow-up.^43^

Once again, Omicron has potentially changed the picture; one preprint study provides population-level evidence to suggest that ‘the Omicron variant is associated with substantial ability to evade immunity from prior infection’ of a sort that was not observed in prior waves, with an estimated HR for reinfection versus primary infection between 1 November and 27 November 2021 versus wave 1 of 2.39.^47^ Another study suggests that the effectiveness of previous SARS-CoV-2 infection in preventing reinfection has fallen from 90.2% against the Alpha variant to 56% against the Omicron variant. However, protection from prior infection against severe outcomes from Omicron remained robust, with a suggested effectiveness of 87.8%.^48^ Furthermore, in addition to evidence suggesting that Omicron may lead to less severe disease,^49^there are also data to suggest that Omicron may also have the ability to evade vaccine-induced immunity, as mentioned above.^50^ To reiterate, if this vaccine evasion is significant, then this calls into question the justification of vaccine mandates as a whole. For our purposes here though, we shall simply note that an early rapid analysis from the SIREN study suggests that protection against infection afforded by natural immunity may be at least as good as that afforded by the two vaccine doses that are deemed sufficient to satisfy vaccine requirements in many jurisdictions. The rapid analysis suggests that the rate of Omicron in those who had received two vaccinations was 73.4 infections (per 10 000 person days), compared with only 60.9 infections (per 10 000 person days) in those who were unvaccinated but had evidence of prior infection. Notably though, this fell to 41.6 per 10 000 in those who had received three vaccine doses.^51^

There are still significant gaps in our understanding of the respective strength and durability of natural and vaccine-induced immunity, as well as the implications that the Omicron variant (and future virus variants) may have. Our aim here is not to settle the scientific debates on this point. However, we believe that it is a fair reflection of the evidence base to say that when vaccine mandates were initiated in 2021 prior to the emergence of Omicron, policy-makers lacked clear and convincing scientific evidence that immunisation is significantly more likely to achieve the relevant public health benefits than natural immunity. At the time of writing, we still lack such data. Studies currently under way may provide clear and convincing evidence of this sort and might change our analysis. However, on the basis of existing data, it is plausible that naturally acquired immunity may be as good as the degree of vaccine-mediated immunity required by proposed mandates. We now turn to the ethical implications of this.

## The case for natural immunity exemptions

The basic case for allowing natural immunity exemptions to vaccination requirements can be outlined straightforwardly. Vaccine requirements have significant costs; they represent a substantial infringement of individual liberty, and there are non-trivial risks associated with vaccination. Moreover, in professional contexts (eg, in hospitals), there are real potential concerns about the downstream effects of such mandates (eg, on patient care through compromised staff numbers). Finally, as detailed above, critics have raised concerns about the harms of discrimination and the exacerbation of existing inequalities that such policies may involve, particularly given the lower vaccine uptake in socially disadvantaged groups.^9 52^ Given all of these different costs, vaccine requirements can only ever be said to be justified if they are *necessary* for achieving a proportionate public health benefit. But if we do not have clear evidence that immunisation is significantly more likely to reduce the public health burden of the virus than natural immunity, then vaccine mandates for the immune are not necessary. It is therefore not justified to mandate the vaccination of those with natural immunity. Furthermore, treating vaccinated individuals differently from those with natural immunity (eg, for the purposes of employment) is discriminatory if there is no material difference in thepublic health risk they pose.

Of course, it is important to establish that such a policy is practically feasible as well as ethically warranted in a theoretical sense. A natural immunity exemption to a vaccine requirement would require ascertaining sufficient proof of natural immunity. As detailed above, a number of vaccine passport schemes already allow for exemptions of this sort. Proof of natural immunity might include proof of a recent positive PCR test result, confirming prior infection within a period for which we have good evidence to suggest that natural immunity endures. Alternatively, more robust proof of immunity could be provided by a serological test result confirming the presence of neutralising antibodies at the time of testing. Looking forward, it might also be possible to use T-cell testing for this purpose; indeed, the Food and Drug Administration (FDA) in the USA has issued an emergency use authorisation for a test that aims to identify an adaptive T-cell immune response to SARS-CoV-2.^53^ One benefit of such testing over antibody testing is that T-cell immune responses appear to endure for longer periods than antibody responses.^53^

It is likely that natural immunity to COVID-19 wanes over time; indeed, there is evidence to suggest that naturally acquired antibodies diminish over time (although T-cell responses appear to be more robust).^38^ Because of that, an individual’s proof of natural immunity should only be deemed valid for a limited period of time. This would need to be regularly revisited, in light of emerging evidence about the duration of natural immunity. However, in view of the aforementioned evidence suggesting the waning of vaccine-induced immunity, the same broad point is true of vaccination; a time limit should be adopted for policies requiring vaccine-induced immunity, based on the likely endurance of such immunity.

### Necessity and uncertainty

One important and complicating factor in responding to a novel infectious threat is the challenge of empirical uncertainty and rapidly changing evidence. It is possible that vaccine mandates were designed at a time when the evidence about the protective benefit of natural immunity was insufficient. At that time, there may have been clear evidence of the benefit of vaccines and uncertainty about the relative protection offered by prior infection. Perhaps that justified an initial assumption that vaccines were superior. However, at this point in the pandemic, that is no longer the case, as significant evidence has accumulated about the about the impact of prior infection. That evidence does not establish that there is a high risk of reinfections leading to severe outcomes in the short-medium term—quite the opposite.

There remains some uncertainty about the relative protection (including particularly the duration of protection) of each form of immunity against COVID-19. A key ethical question is how we should respond to that uncertainty and what evidence we take as being sufficient. We have suggested that in the absence of clear evidence that vaccine-induced immunity is superior, governments should permit mandate exemptions for those with natural immunity. However, it might be argued that this standard is too low; perhaps it could instead be claimed that exemptions are only justified if *we* can prove that vaccinating those with natural immunity is definitely *not* necessary. This is a subtle change but a crucial one. Although we believe that the evidence base cannot establish that a vaccine mandate is necessary in those with natural immunity, we do not yet have sufficient evidence about the differences between natural and vaccine-induced immunity to prove that vaccines in this group are definitely *not* necessary.

It might be argued that the higher standard of proof is more appropriate in the context of a pandemic due to the high stakes involved. If we adopt the higher standard, we might minimise the chance of unwittingly allowing people who are actually infectious to spread the virus. Given the clear need to protect the vulnerable, it might be argued that we should exercise precaution by invoking the higher standard of proof.

The problem with taking this sort of precautionary approach is that it overlooks the significant harms that such precaution has in this context. This is a common criticism of precautionary approaches across a wide range of contexts (including public health),^54^ but the harms of precaution in a pandemic can be particularly salient.^55^ There are a considerable number of individuals who remain reluctant to receive a vaccination. For instance, in the USA, a report published in August 2021 by an advisory body to the US Department of Health and Human Services suggestedthat 30% of the adult population are unvaccinated, and that only approximately 44% of that unvaccinated group would be willing to receive a vaccination.^56^ When unwilling individuals are subject to a vaccine mandate, their liberties are significantly restricted. In the case of professional mandates, people’s employment and income are at stake. That is not to say that such requirements cannot be justified. Rather, the point we are making here is that to assume that the higher standard of proof is correct is to assume that avoiding an uncertain (but very likely low) risk (ie, of an increased public health burden attributable to unvaccinated individuals with natural immunity) should take precedence over avoiding the known and quantifiable harms of restricting individual liberties in this way. But that seems ethically fraught. In the absence of compelling evidence that vaccine-induced immunity is significantly more likely to reduce public health burdens than natural immunity, we believe that the case for ethical necessity cannot be convincingly made. Vaccine requirements for those with natural immunity may unnecessarily (i) restrict liberty, (ii) expose individuals to risks of harm, and (iii) expose institutions to bad downstream effects, all without the corresponding promise of proportionate benefit. Furthermore, they may use a scarce resource that is needed elsewhere; vaccines remain scarce in many countries where vulnerable individuals are yet to receive a single dose. The burden lies with those claiming that there is an increased public health risk associated with exemptions for naturally immune individuals to show that this is indeed the case.

### Pragmatic issues

Nonetheless, even if the case for necessity is not convincing, it might be argued that there are some important practical reasons for refraining from allowing natural immunity exemptions. Earlier in the pandemic, critics of immunity passports raised the concern that they could incentivise people to intentionally become infected with the coronavirus, and that this could have particularly disadvantageous effects on some of the most vulnerable groups in society who remain suspicious of vaccines.^52^ Indeed, in early 2022, there was a widely reported case of a Czech singer tragically dying after intentionally contracting COVID-19 in order to obtain a natural immunity exemption to her country’s vaccine mandate.^57^

Second, proof of vaccination status is simple, binary and verifiable, while natural immunity is difficult to monitor, meaning that natural immunity exemptions would be complex and require additional resources. Third, the positive predictive value of the tests we might accept as proof of natural immunity may vary, depending on both the tests employed and the prevalence of infections at the time and place they are deployed.^58^

We believe that these objections have limited force. With respect to the first objection, while recognising the significance of anecdotal reports of the phenomenon, we should also be wary of extrapolating just how prevalent it may be in comparison to the large numbers of people whose liberty may be being unnecessarily restricted if natural immunity exemptions are not permitted.^59 60^ Rather than providing a basis for rejecting natural immunity exemptions, we believe that these tragic cases illustrate the paramount importance of engaging with vaccine sceptics to educate them about the pitfalls of acquiring immunity via natural infection. It is also important to acknowledge that we are now at a very different stage of the pandemic. Billions of people have now been vaccinated and would have no need for a natural immunity exemption. Moreover, in many places at least, unvaccinated people in 2022 are afforded far more freedoms today than they were afforded in 2020.

Furthermore, allowing exemptions for natural immunity might be more consistent with a concern for social justice. Data suggest that vaccine uptake is lower in marginalised groups,^61 62^ and they will be disadvantaged by those requirements that recognise *only* vaccine-induced immunity. In addition, as Patel *et al* highlighted, a number of factors led low SES (socioeconomic status) groups to have greater exposure to the virus over the course of the pandemic,^63^ and there are data to suggest that deprived areas have seen more confirmed cases of COVID-19.^64^ Denying the protective effect of natural immunity in vaccine requirements puts these lower SES groups at a disadvantage. Nonetheless, it might finally be argued in this vein that allowing natural immunity exemptions would amount to ‘giving up’ on the idea of educating vaccine sceptics, and leaving them to the danger of acquiring immunity via natural infection. However, it is not clear that natural immunity exemptions must have this connotation if they are paired with a commitment to continued education initiatives, and strong public health messaging about the superiority of acquiring immunity via vaccination rather than natural infection. It might be true that in some circumstances, public officials can be justified in prohibiting one option to promote uptake of an option that is superior in some sense; but in this case, prohibiting one option would significantly infringe rights and expose individuals to harm. It is not clear that the benefits of simply increasing vaccine uptake in sceptical groups (as opposed to the wider public health benefits that mandates aim to achieve) are sufficient to justify these costs.

With respect to the second objection, it is clearly important to ensure that both the data regarding the protective effect of natural immunity and the standards of proof that we accept for natural immunity are sufficiently robust. With respect to the former, we refer back to the second section of this paper. With respect to the latter, we should only accept positive viral detection test results as proof if the tests meet acceptable levels of sensitivity and specificity, and have an acceptable positive predictive value given prevalence of the virus at the time. It may also be possible to ensure more robust protection against false positive results by adopting serial testing regimes. Alternatively, it might be argued that natural immunity exemptions should only be issued on the basis of more robust direct proof of natural immunity with serological evidence of neutralising antibodies, or potentially T-cell testing. Where more robust forms of testing are associated with significant cost, the cost of testing might be passed on to the individual, or to the employer.

In any case though, the practicality of using natural immunity as sufficient proof of an individual’s low public health risk has already been demonstrated by existing vaccine certificate schemes such as those in Italy and Israel. In both countries, it is possible to obtain a pass with a certificate of recovery issued by a state health authority showing proof of a recent positive COVID-19 test result. Furthermore, in Israel, those who have tested positive on a serologic test can receive a pass if, having not been vaccinated prior to testing, they have since received at least one dose of a COVID-19 vaccine.^13^

### Political considerations

One significant reason, we suspect, for not considering natural immunity as equivalent to vaccine-acquired immunity for the purposes of vaccine certification is the concern that this would provide support for those opposed to vaccination, or undermine public health messages about the importance and benefits of having the vaccine.^65^ Notably, after he attempted to enter the country with proof of recent recovery from COVID-19, Australian immigration authorities cancelled Novak Djokovic’s visa under powers authorising the deportation of anyone who is a potential risk to ‘the health, safety or good order of the Australian community’.^66^

It is possible that natural immunity exemptions would embolden anti-vaccination movements. However, we suggest that being clear (as we have tried to do at the start of this paper) about different types of natural immunity claims would help redress any misinformation or misunderstanding. There is an important distinction between arguing that natural immunity, once acquired, may confer sufficient protection (which we have made) and the claim that natural immunity is a good way to acquire immunity (which we do not support, and have not made). Second, even if it were the case that public health messages could be misinterpreted (if mandates allowed natural immunity exemption), we would argue that it would be deeply ethically problematic to mandate vaccination of the immune for that reason. It would imply that (for example) care workers with natural immunity who pose little risk were being used as a means to prevent wider misunderstanding in the community.

Throughout the pandemic, there has been reluctance to consider natural immunity as protective against COVID-19, perhaps partly due to concerns about incentivising deliberate infection, and uncertainty about the strength and durability of natural immunity. However, legislators now cannot avoid the issue for two reasons: first, there is less reason to believe people will flood to be infected when there is the safer alternative of vaccination, and second, there is now evidence to suggest that those with immunity from natural infection appear to pose a low risk to others, comparable to individuals with vaccine-induced immunity.

## Conclusion

There still some gaps in our understanding of the differences between natural and vaccine-induced immunity. For some, this uncertainty might lend support to a ‘belt and braces’ approach of ensuring that those with natural immunity also acquire vaccine-induced immunity. However, as we have argued, there are significant ethical costs with a precautionary strategy of mandating this. Our argument in this paper is based on a claim about the current evidence of the relative protection of vaccine-induced versus naturally acquired immunity against COVID-19, as it pertains to vaccine requirements that came into force in 2021. This means that if the evidence changes, our conclusions will also change. For example, if further studies are published that clearly show that vaccination is superior for reducing transmission of the virus, or that naturally acquired immunity wanes substantially over time, then that would (other things being equal) support a vaccine mandate in this group (potentially at least after a certain period has elapsed following infection). With some studies estimating that reinfection is likely to become increasingly common as the pandemic continues,^67^ it is crucial that infections among those with both natural and vaccine-induced immunity are closely monitored.

At the present time, however, it appears that requiring those with natural immunity to undergo vaccination is not clearly necessary to obtain a substantial public health benefit. If so, it is discriminatory to treat natural immunity differently to vaccine-mediated immunity, and this is something that ethical, evidence-based public health policy should reflect. This argument implies that if governments continue with mandates, they should allow (for example) healthcare workers with sufficient proof of natural immunity to continue working for as long as that immunity can reasonably be expected to endure. Such an exemption would prevent the unnecessary loss of valuable workers who do not pose an increased risk to vulnerable patients and residents.

## Data Availability

There are no data in this work.
